# Fabrication of a Covalent Triazine Framework Functional Interlayer for High-Performance Lithium–Sulfur Batteries

**DOI:** 10.3390/nano12020255

**Published:** 2022-01-14

**Authors:** Ben Hu, Bing Ding, Chong Xu, Zengjie Fan, Derong Luo, Peng Li, Hui Dou, Xiaogang Zhang

**Affiliations:** 1 Jiangsu Key Laboratory of Electrochemical Energy-Storage Technologies, College of Materials Science and Technology, Nanjing University of Aeronautics and Astronautics, Nanjing 210016, China; 1105786873@nuaa.edu.cn (B.H.); xuchong@nuaa.edu.cn (C.X.); zengjiefan@nuaa.edu.cn (Z.F.); dr.luo@nuaa.edu.cn (D.L.); lpeng@nuaa.edu.cn (P.L.); dh_msc@nuaa.edu.cn (H.D.); 2 Shenzhen Research Institute, Nanjing University of Aeronautics and Astronautics, Shenzhen 518000, China

**Keywords:** Li–S batteries, shuttling effect, covalent triazine framework, functional interlayer, polysulfide retention

## Abstract

The shuttling effect of polysulfides is one of the major problems of lithium–sulfur (Li–S) batteries, which causes rapid capacity fading during cycling. Modification of the commercial separator with a functional interlayer is an effective strategy to address this issue. Herein, we modified the commercial Celgard separator of Li–S batteries with one-dimensional (1D) covalent triazine framework (CTF) and a carbon nanotube (CNT) composite as a functional interlayer. The intertwined CTF/CNT can provide a fast lithium ionic/electronic transport pathway and strong adsorption capability towards polysulfides. The Li–S batteries with the CTF/CNT/Celgard separator delivered a high initial capacity of 1314 mAh g^−1^ at 0.1 C and remained at 684 mAh g^−1^ after 400 cycles^−1^ at 1 C. Theoretical calculation and static-adsorption experiments indicated that the triazine ring in the CTF skeleton possessed strong adsorption capability towards polysulfides. The work described here demonstrates the potential for CTF-based permselective membranes as separators in Li–S batteries.

## 1. Introduction

Li–S batteries have been recognized as high-energy electrochemical energy-store devices because of the high theoretical energy density (2600 Wh kg^−1^) and low cost of elemental sulfur [1]. The practical application of Li–S batteries, however, has been severely hindered by their several inherent problems. One of the serious issues is the shuttling effect caused by the dissolution of the polysulfide intermediates in the organic electrolyte. During the charge–discharge cycles, the polysulfide intermediates will shuttle across the separator and react with the lithium anode, resulting in rapid capacity loss and low Coulombic efficiency of the sulfur cathode [2,3,4,5,6]. To reduce the shuttling effect of polysulfides, tremendous efforts have been devoted to developing nanostructured sulfur hosts, such as carbon-based materials, conductive polymers, transition metal oxides, and their composites [7,8,9,10,11,12,13]. These sulfur–host materials can enhance the polysulfide retention by physically confining the polysulfide intermediates in the pores. During the long charge/discharge cycles, however, the dissolution of polysulfides in the electrolyte cannot be completely avoided. Recent modification of the separator with a functional interlayer has been demonstrated to be another effective strategy to improve the performance of Li–S batteries [14,15]. The interlayer can serve as a physical barrier against the polysulfides without affecting the transport of lithium ions. In addition, the functional groups on the interlayers and their polar surfaces can anchor the polysulfides based on chemical interaction [16,17]. The use of an interlayer in Li–S batteries therefore can effectively improve the utilization of sulfur and slow down the capacity fading [18,19,20]. However, the addition of interlayers with high weight in the battery will definitely compromise the gravimetric energy density of the full cell. Therefore, designing light-weight interlayers with strong capture ability towards polysulfides is highly desired for achieving high-performance Li–S batteries. 

Since first reported by Yaghi et al. in 2005, covalent organic frameworks (COFs) have been widely studied in various fields [21]. Due to their high porosity, well-defined pore structure, tunable pore size, and designable chemical structure, COFs are promising in ion transport and ion sieving application [22,23,24,25]. With the above-mentioned advantages, COF materials have been investigated as the sulfur host to prepare COF/S composite cathodes for Li–S batteries [26,27]. For example, Tang et al. reported that when the boronate ester COFs were used as a sulfur host, the positively polarized B atoms and negatively polarized O atoms in the COF skeleton exhibited strong adsorption capability towards the polysulfide intermediates, which could effectively reduce the shuttling effect. The assembled Li–S battery exhibited a high initial capacity of 1628 mAh g^−1^ and retained 929 mAh g^−1^ after 100 cycles [26]. However, most COFs possess very low electronic conductivity. The use of COFs as sulfur hosts will slow down the reaction kinetics of sulfur, leading to the reduction in the rate capability of the Li–S batteries [28,29,30,31,32]. Therefore, more attention has been paid to applying COF materials in Li–S batteries as interlayer materials [33,34,35,36,37].

Herein, we modified the commercial Celgard separator of Li–S batteries with a one-dimensional (1D) covalent triazine framework (CTF)-type COF and 1D carbon nanotube (CNT) composite as a functional interlayer. The intertwined CTF/CNT composite can provide a fast lithium ionic/electronic transport pathway and demonstrates strong adsorption capability towards polysulfides. When the CTF/CNT/Celgard separator was applied in Li–S batteries, the rate capability and cycle performance of the Super P/S cathodes were markedly enhanced. After 400 charge–discharge cycles at a current density of 1 C, the Super P/S cathode still exhibited a high capacity of 684 mAh g^−1^ with an average Coulombic efficiency of 98.4%. Even at a high mass loading of sulfur (2 mg cm^−2^), the Li–S battery using the CTF/CNT/Celgard separator still demonstrated much better performance compared to the Li–S battery using the Celgard separator. This work not only demonstrates the potential of COF materials in Li–S battery applications, but also provides new insights into the structural/chemical design of permselective materials for retaining the active materials in electrochemical devices.

## 2. Experimental Section

### 2.1. Preparation and Characterization

Synthesized TpMA CTF: 1,3,5-triformylphloroglucinol (Tp, 42.03 mg, 0.2 mmol) and melamine (Me, 25.2 mg, 0.2 mmol) were added to a Pyrex tube containing 4 mL of dimethyl sulfoxide (DMSO) and 0.4 mL of acetic acid (6 mol L^−1^). After ultrasonication for 20 min, the tube was degassed three times by liquid nitrogen freeze–pump–thaw cycles. Then, the tube was sealed and heated at 120 °C for 72 h. The products were washed three times with DMSO, dimethylformamide (DMF), anhydrous acetone, and water alternatively to remove the unreacted products. Then, the COF powder material (~50.4 mg) was obtained after drying at 100 °C for 12 h under vacuum. The yield was ~75%.

Preparation of the CTF/CNT-modified Celgard separator: Typically, 1 mL of CNT dispersion (1 mg mL^−1^), 8 mg of CTF powders, and 1 mg of polyvinylidene difluoride (PVDF) were dispersed in 60 mL of DMF solution and ultrasonicated for 6 h to form a homogeneous suspension. The suspension was deposited onto the commercial Celgard polypropylene (PP) separator via vacuum-assisted filtration. The CTF/CNT-modified separator was dried at room temperature for 12 h and further dried under vacuum at 60 °C for another 12 h. Subsequently, the CTF/CNT-modified Celgard separators were cut into circular disks with a diameter of 19 mm. The loading mass of the CTF/CNT composite on the Celgard separator was approximately 0.51 mg cm^−2^. The CNT-modified Celgard separator was also fabricated as the control sample using the same procedures except replacing the CTF with a CNT of the same weight.

### 2.2. Electrochemical Measurement

Super P conductive carbon black (300 mg) and 700 mg of sulfur powder were mixed by planetary ball milling for 2 h. Then, the mixture was transferred to a 20 mL glass ampoule. After sealing, the glass ampoule was heated at 155 °C for 10 h to obtain Super P/S composite. The Super P/S composite was accurately weighed with conductive carbon (Super P) and PVDF with a mass ratio of 8:1:1 and added with an appropriate amount of N-methylpyrrolidone (NMP) for grinding into a uniform and viscous slurry. Subsequently, the slurry was coated on carbon-coated aluminum foil and dried in a vacuum drying oven at 60 °C for 24 h. Finally, the dried electrode was cut into a circular plate with a diameter of 12 mm. Electrochemical performances were investigated using a CR2032 coin-type cell; 1.0 M LiTFSI in a mixed solvent of DOL and DME (volume ratio 1:1) with 1% LiNO_3_ additive was used as the electrolyte. The Super P/S electrode and metal lithium metal were used as the cathode and anode, respectively. CTF/CNT-modified Celgard 2500, CNT-modified Celgard separator or pristine Celgard was used as the separator. Galvanostatic discharge/charge measurements were performed with a LANDTE CT2001A battery test system in the voltage range of 1.7–2.8 V. Cyclic voltammetry (CV) and electrochemical impendence spectroscopy (EIS, in the frequency range of 10^6^ Hz to 10^−1^ Hz) were conducted on a Biologic VMP-3 potentiostat.

## 3. Results and Discussion 

**Figure 1:**
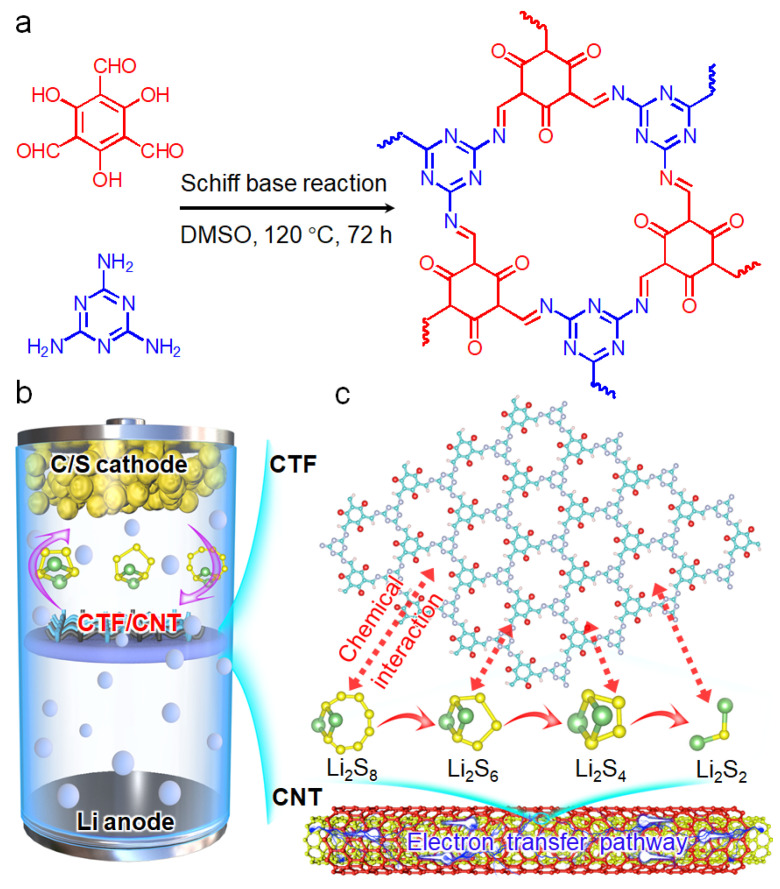
(**a**) Synthesis procedure of CTF based on a Schiff base reaction. Schematic illustration of (**b**) the Li–S cell using the CTF/CNT/Celgard separator and (**c**) the structure of the CTF/CNT composite and its interaction with polysulfides.

The 1D TpMA CTF nanorod was synthesized based on a Schiff base reaction of Tp and Me (Figure 1a). A CTF/CNT/Celgard separator was prepared by vacuum filtering the mixed dispersion of CTF, CNT, and PVDF on the commercial Celgard separator, which was then used for assembling the Li–S batteries (Figure 1b). It is noted that without using CNT, the CTF powders easily dropped off the separators. As schematically displayed in Figure 1c, the triazine ring in the CTF can interact with polysulfides, therefore preventing the shuttling of polysulfides. CNT is well mixed with the CTF to improve the electron transfer during the redox reaction of polysulfides. In addition, the CNT layer is tightly combined with the sulfur cathode, which can work as a current collect to improve the electron conductivity of the cathode. 

**Figure 2:**
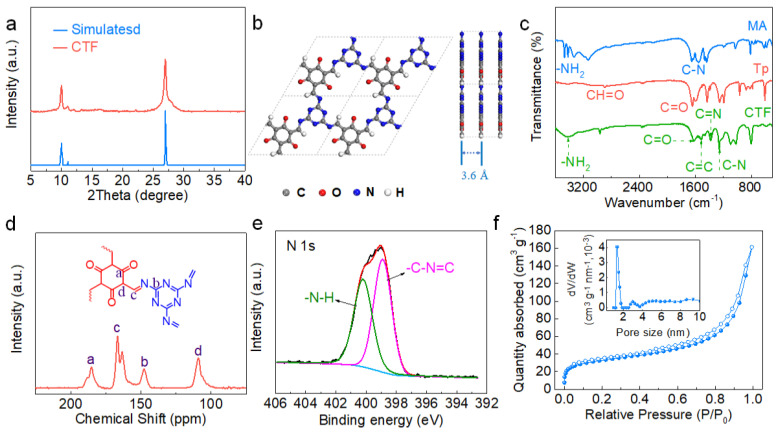
Structural characterization of the CTF: (**a**) XRD of experimental pattern and simulated pattern; (**b**) Structural models of the CTF with quasi-AA stacking; (**c**) FT-IR spectra of the CTF and precursor; (**d**) ^13^C CP/MAS solid-state NMR spectra; (**e**) High-resolution N *1s* XPS spectra; (**f**) N_2_ adsorption–desorption isotherms of the CTF (insets: pore size distribution curve).

The chemical structure of the CTF was first characterized. As shown in the XRD pattern of the CTF (Figure 2a), two diffraction peaks at ~9.9° and 27.1° can be attributed to the (100) and (002) planes, respectively [38]. From the position of the characteristic peak of (001) plane, the layer distance was calculated to be 3.5 Å. Figure 2b shows the simulated structure of the CTF constructed along the *c*-axis direction and the *b*-axis direction. The layers of the CTF are stacked through π-π interaction, and the hexagonal holes on each layer are piled up along the *c*-axis direction. The simulated interlayer distance is ~3.6 Å, which is very close to the value calculated from the XRD pattern. The functional group and chemical position of the CTF were confirmed by FT-IR, ^13^C NMR, and XPS spectra. In the FT-IR spectrum, the peaks at 1628, 1517, and 1253 cm^−1^ correspond to the stretching characteristic absorption peaks of C=O, C=C, and C–N, respectively, indicating the formation of carbonyl and triazine units in the CTF skeleton (Figure 2c). The disappearance of the peak representing the stretch of NH2 in the structure demonstrated the complete conversion of this reaction [39]. The characteristic peaks in the ^13^C NMR spectrum reflect the carbon chemical environment at different positions (Figure 2d). The signal at 185.1 ppm can be ascribed to the ketone carbonyl C=O while the peaks at ~147.3 and 109.1 ppm are attributed to C-N and C=C, respectively. The peaks at 167 and 163 ppm are the characteristic peaks of triazine rings in CTF [40]. The XPS survey of the CTF indicated the existence of C, N, and O (Figure S1a). In the high-resolution N *1s* spectra, two peaks located at 398.87 and 400.2 eV are assigned to the triazine group (C−N=C) and –N−H group, respectively (Figure 2e) [41]. The C *1s* XPS spectrum was fitted by three peaks located at 284.5, 286.6, and 288.5 eV, which are attributed to the carbon atoms in C=C, C=O, and the triazine ring (N−C=N), respectively (Figure S1b) [41,42,43]. The N_2_ adsorption–desorption isotherms of the CTF display a type-I curve. The BET specific surface area was calculated to be 111.0 m^2^ g^−1^. The pore size distribution curve indicates a pore size centered at 1.5 nm (Figure 2f). The TGA curve shows that the thermal stability of the CTF reaches 400 °C (Figure S2).

**Figure 3:**
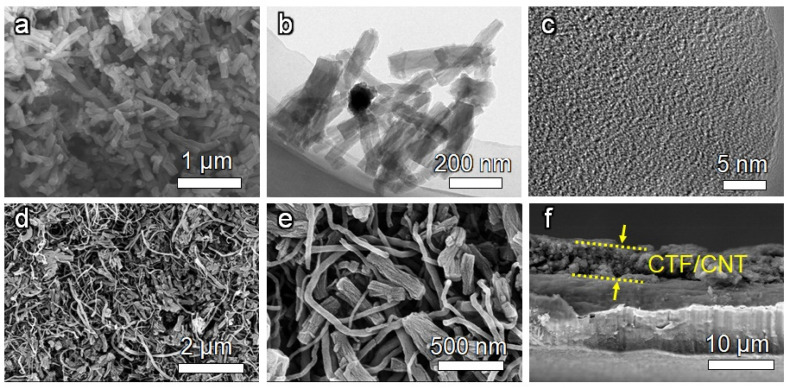
(**a**) SEM image, (**b**) TEM image, and (**c**) HRTEM image of the CTF. (**d,e**) Top-view SEM images and (**f**) cross-sectional SEM image of the CTF/CNT/Celgard separator.

The morphology of the CTF was investigated by electron microscopy. Scanning electron microscopy (SEM, Figure 3a) and transmission electron microscopy (TEM, Figure 3b) images of the CTF indicate the nanofiber architecture with a diameter of ~100 nm. From the high-resolution TEM (HRTEM) images (Figure 3c), a lamellar structure can be observed, which is formed by the π-π stacking of the CTF layer. 

The CTF/CNT/Celgard separator was prepared by filtering the mixed suspension of the CTF and CNT on the commercial Celgard separator. PVDF binder was also added for fixing the CTF and CNT on the separator. For comparison, the CNT-modified separator was also prepared by replacing the CTF with the CNT (Figure S3). Before coating the interlayer material, the Celgard separator had porous and fibrous morphology (Figure S3a,b). After being modified by CTF/CNT, SEM images and energy-dispersive X-ray spectroscopy (EDS) mapping showed that the surface of the separator was homogeneously covered by the CTF and CNT, which were interwined with each other (Figure 3d,e and Figure S4). Cross-sectional SEM images indicated that the thickness of the CTF/CNT interlayer is about 5 µm (Figure 3f). 

The contact angle of the Li–S electrolyte on the surface of the Celgard, CNT/Celgard, and CTF/CNT/Celgard interlayer was measured to compare the electrolyte wettability on different separators. As shown in Figure S5, the CTF/CNT/Celgard separator shows a much smaller contact angle (7.44°) compared to the CNT/Celgard separator (15.63°) and pristine Celgard separator (26.69°). The lower contact angle means better wettability towards the electrolyte, which will reduce the interface impedance between the interlayer, electrolyte, and cathode as well as facilitate the ion diffusion through the separator. To verify the effect of the CTF/CNT in promoting the lithium ion migration, a lithium–lithium symmetric battery was assembled, and a current-time (*i*-*t*) curve was constructed to calculate the lithium ion migration. As shown in Figure S6, lithium ion migration in the CTF/CNT/Celgard separator was calculated to be 0.634, which was significantly higher than that in the CNT/Celgard separator (0.538) and Celgard separator (0.325), indicating that the CTF/CNT composite can promote lithium ion migration. 

**Figure 4:**
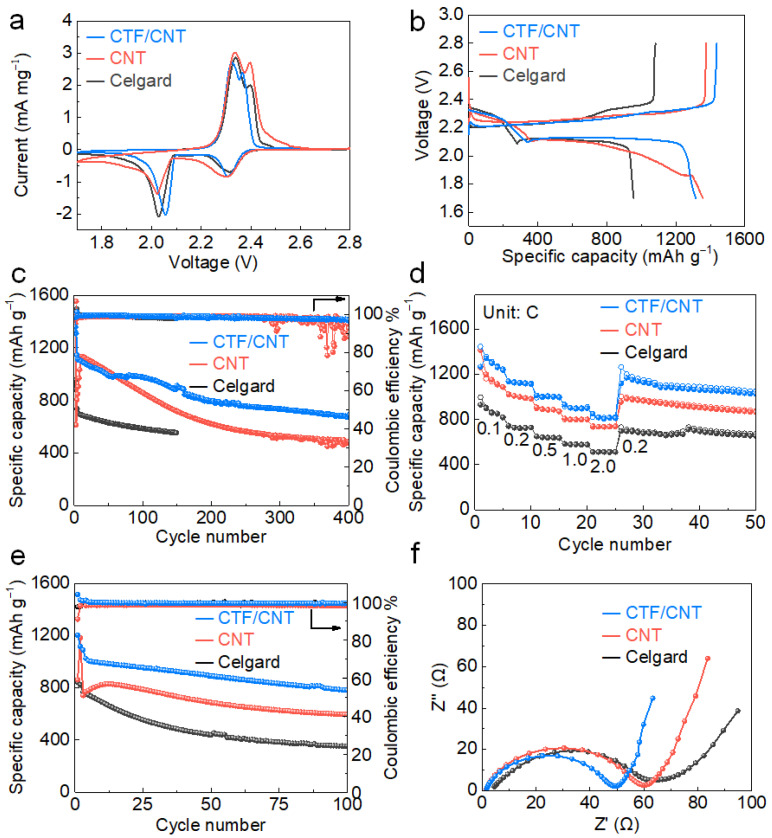
Electrochemical performances of Li–S cells with different separators: (**a**) CV curves at 0.1 mV s^−1^, (**b**) initial galvanostatic discharge–charge profiles at 0.1 C, (**c**) cycling performance at 1 C, and (**d**) rate performance, (**e**) cycling stability of Li–S cells with a high mass loading of sulfur (2 mg cm^−2^) at 0.5 C, and (**f**) EIS plots after electrochemical cycling.

To evaluate the effect of the CTF/CNT/Celgard separator on the electrochemical performance of Li–S batteries, 2032-type cells were assembled using Super P/S composite as the cathode material. The sulfur content in the Super P/S cathode was ~69 *wt.*% (Figure S7), and the mass loading of sulfur on the electrode was about 1.2 mg cm^–2^. For comparison, Li–S batteries using pristine Celgard separator and CNT/Celgard separator were also assembled. Figure 4a shows the cyclic voltammetry (CV) curves of all cells measured within a potential range of 1.7–2.8 V and at a scan rate of 0.1 mV s^−1^. All CV curves exhibited typical characteristics of the electrochemical redox reaction of sulfur. The two reduction peaks were attributed to the conversion reaction from solid cyclic S_8_ molecules to soluble long-chain Li_2_S_n_ (4 ≤ n ≤ 8) and further conversion to insoluble Li_2_S_2_/Li_2_S. The oxidation peak corresponds to the reaction process of insoluble Li_2_S_2_/Li_2_S converting to solid S8 [43,44,45]. Compared with the cells using the Celgard separator, the cell using the CTF/CNT/Celgard separator exhibited higher reduction voltage and lower oxidation voltage, indicating the faster redox kinetics promoted by the CTF/CNT composite. The cell using the CNT/Celgard separator had lower current density compared to the battery using the Celgard separator This was because the cell using the CNT/Celgard separator requires activation in the initial cycles (Figure 4e). To further investigate the effects of the modified separator in improving the reaction kinetics, CV curves at different scan rates (*v*) were further measured. The lithium ion diffusion coefficient of Li–S batteries using the CTF/CNT/Celgard, CNT/Celgard, and Celgard separators was calculated based on the Randles–Sevick formula to reveal the difference in the reaction kinetics (Figure S8a–c). The peak current (*I*_p_) and *v*^1/2^ of different cells at different stages of reaction (i.e., α, β, γ peaks) were plotted and linearly fitted to obtain the slope value (Figure S8d–f). The battery using the CTF/CNT/Celgard separator had a comparable lithium ion diffusion coefficient with the battery using the Celgard separator (Table S1), indicating that the CTF/CNT interlayer does not hinder the diffusion of lithium ions. All of these results indicate that the CTF/CNT interlayer promotes the reaction dynamic of the Li–S battery without affecting the lithium ion diffusion. The initial galvanostatic charge–discharge curves of Li–S batteries using different separators are compared in Figure 4b. CTF/CNT modified separator showed the lowest overpotential, which was consistent with the results of CV. Figure 4c describes the cycle performances of the Li–S cells, which were pre-cycled at 0.1 C for three cycles before cycling at 1 C. The initial capacity of the cell with the CTF/CNT/Celgard separator was 1314 mAh g^−1^, while the initial capacity of the cells with the CNT/Celgard separator and Celgard separator was 1356 and 955 mAh g^−1^, respectively. At 1 C, the initial capacity of the cell with the CTF/CNT/Celgard separator was 1145 mAh g^−1^ and remained at 684 mAh g^−1^ after 400 cycles. The average Coulombic efficiency and capacity decay per cycle were 98.4% and 0.1%, respectively. In contrast, the capacity of the battery using the CNT/Celgard separator was 484 mAh g^−1^ after 400 cycles. The average Coulombic efficiency was 95.2%. The capacity of the cell with the Celgard separator rapidly decreased to 551 mAh g^−1^ after 150 cycles. The average Coulombic efficiency during the 150 cycles was 98.8%. The battery using the CTF/CNT/Celgard separator showed the best cycle stability because the polysulfide shuttling was attenuated. The capacity and cycle performance of Li–S battery using the CTF/CNT/Celgard separator were also superior to the Li–S batteries using various functional interlayers (Table S2).

The rate performance reflects the redox reaction kinetics of Li–S chemistry. At current densities of 0.1, 0.2, 0.5, 1, and 2 C, the average discharge capacities of the Li–S battery using the CTF/CNT/Celgard separator reached 1280, 1122, 1001, 905, and 819 mAh g^−1^, respectively (Figure 4d). When the current density was switched back to 0.1 C, the average discharge capacity returned to 1140 mAh g^−1^. The average discharge capacities of the cells with the CNT/Celgard separator and Celgard separator at 2 C quickly decreased to 737 and 525 mAh g^−1^, respectively. With the increasing current density from 0.2 to 2 C, the typical characteristic plateaus of the sulfur cathode were identified (Figure S9), implying the smooth redox reaction of sulfur species at high current densities [44]. The cycling performance of Li–S batteries using different separators at a high mass loading of sulfur (2 mg cm^−2^) was also measured (Figure 4e). The initial discharge capacity of the Li–S battery using the CTF/CNT/Celgard separator reached 1090 mAh g^−1^ at 0.5 C after activation at 0.1 C for two cycles. After 100 cycles, the capacity still remained at 782 mAh g^−1^. Both of the initial capacity and retention rate after cycling of the battery using the CTF/CNT/Celgard separator were higher than those of batteries using the CNT/Celgard and Celgard separators. The improved cycling performances and rate capability the battery using the CTF/CNT/Celgard separator can be attributed to the reduced shuttling effect of the lithium polysulfide and the improved redox kinetics. The improved electrochemical kinetics was also revealed by the EIS measurement measured after 100 cycles. As shown in Figure 4f, compared to the battery using the Celgard separator, the batteries using the CTF/CNT/Celgard and CNT/Celgard separators had a higher slope in the low-frequency district and smaller semi-circle in the high-frequency district, indicating lower electron transfer and ion-diffusion resistance, respectively [45,46]. This is because the coating of CNT on the separator can accelerate the electron conduction and promote the reaction kinetics of the cathode during cycling. The impedance of the battery using the CTF/CNT/Celgard separator after 100 cycles was significantly lower than those of the batteries using the CNT/Celgard and Celgard separator (Figure 4f).

**Figure 5:**
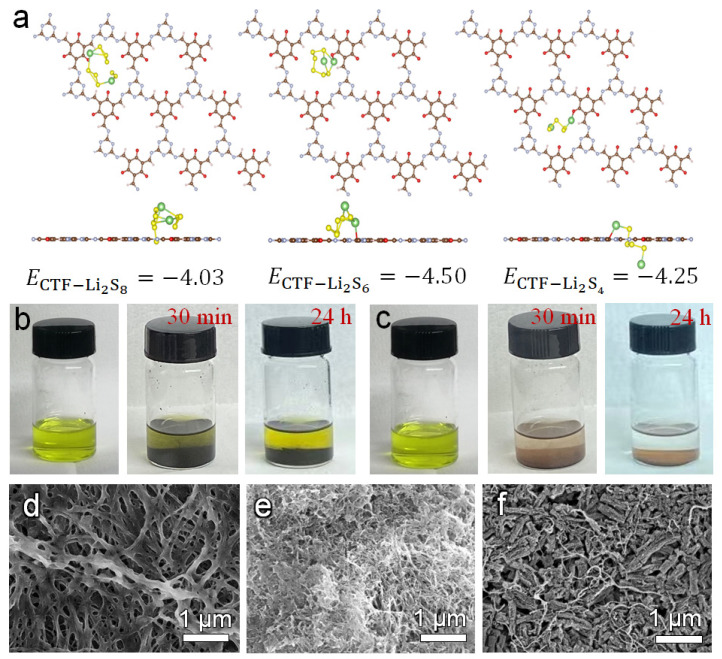
(**a**) DFT calculations of Li_2_S_8_, Li_2_S_6_, and Li_2_S_4_ adsorption on CTF, static-adsorption experiments of (**b**) CNT and (**c**) CTF in a Li_2_S_4_ solution. SEM images of (**d**) the Celgard separator, (**e**) CNT/Celgard separator, and (**f**) CTF/CNT/Celgard separator after cycling.

In order to investigate the mechanism of CTF/CNT in improving the rate and cycling performances of Li–S batteries, first-principle density functional theory (DFT) calculations were conducted to illustrate the adsorption and transformation of polysulfides on the CTF structure skeleton. Figure 5a shows the optimized geometry of Li_2_S_8_, Li_2_S_6_, and Li_2_S_4_, which were adsorbed on the CTF surface. Li_2_S_8_, Li_2_S_6_, and Li_2_S_4_ were anchored at the N sites of the CTF skeleton with a binding energy of −4.03, −4.50, and −4.25 eV, respectively. All of these values are significantly higher than the binding energy on graphene plane [43]. The strong interaction can effectively restrain the diffusion of lithium polysulfide, leading to an improved Coulombic efficiency and cycle stability of Li–S batteries. Static-adsorption measurement using Li_2_S_4_ solution was conducted. As shown in the digital photos (Figure 5b,c), the Li_2_S_4_ solution was dark yellow. After 30 min, the supernatant of the solution with the CTF powder began to become transparent. Even after 24 h, the color of the glass bottle with the CNT powder remained unchanged, indicating that Li_2_S_4_ in the solution can be completely adsorbed by the CTF powder. The results show that CTF powder has excellent adsorption capability toward Li_2_S_4_, which can effectively slow down the shuttle of polysulfides and improve the cycle performance of Li–S batteries. 

The morphology of the CTF/CNT/Celgard, CNT/Celgard, and Celgard separators used in the Li–S batteries after cycling was observed by SEM. As shown in Figure 5f and Figure S10c, the surface CTF/CNT before and after cycling was also same. On the contrary, some large particle were deposited on the fiber of pristine Celgard (Figure 5d and Figure S10a) and on the CNT (Figure 5e and Figure S10b). This is because the lithiophilic sites on the CTF skeleton promote ionic conduction and reduce the shuttling of lithium polysulfide, which prevents the formation of dead sulfur. 

## 4. Conclusions

In summary, 1D–1D architectured CTF/CNT was fabricated for modifying the separator in Li–S batteries. The Li–S batteries using the CTF/CNT/Celgard separator had high sulfur utilization, excellent favorable cycling performance, and high Coulombic efficiency. During the 400-cycle charge/discharge cycling, the average capacity fading and Coulombic efficiency were 0.1% and 98.4%. The results of theoretical calculation and static-adsorption measurement indicated that CTF material with triazine structure dramatically improved the permselectivity of the separator through strong chemical adsorption of polysulfide. Our work not only offers insight into the roles of CTF materials in Li–S chemistry but also provides an effective strategy for the development of practical Li–S batteries with high energy density and a long life.

## Data Availability

The data presented in this study are available on request from the corresponding author.

## Supplementary Materials

The following supporting information can be downloaded at: https://www.mdpi.com/article/10.3390/nano12020255/s1, Experimental section; Figure S1: XPS survey; Figure S2: TGA curve; Figure S3. SEM images of separators; Figure S4: SEM image and EDX mapping; Figure S5: Contact angle measurements; Figure S6. I-t curves and Impedance plots. Figure S7: SEM images, XRD patterns and TGA curve of Super P/S composite. Figure S8: CV curves and plots of peak current vs. square root of scan rates. Figure S9: Galvanostatic discharge-charge profiles. Figure S10: SEM images of the separator after cycling. Table S1: Summary of lithium ion diffusion coefficient; Table S2: Electrochemical performance of Li–S batteries by using various modified separators.
