# Supplementary material for: Fabrication of a Covalent Triazine Framework Functional Interlayer for High-Performance Lithium–Sulfur Batteries
Source: Nanomaterials (Basel). 2022 Jan 14;12(2):255. doi: 10.3390/nano12020255 (PMC8779782; doi:10.3390/nano12020255)
Supplement: Supplementary file 1 [file nanomaterials-12-00255-s001.zip › nanomaterials-1495489-supplementary.pdf]

nanomaterials

Supporting Information

Fabrication of a Covalent Triazine Framework Functional Interlayer for High-Performance Lithium–Sulfur Batteries

Ben Hu [1], Bing Ding [1,2,*], Chong Xu [1], Zengjie Fan [1], Derong Luo [1], Peng Li [1], Hui Dou [1] and Xiaogang Zhang [1,2,*]

[1] Jiangsu Key Laboratory of Electrochemical Energy-Storage Technologies, College of Materials Science and Technology, Nanjing University of Aeronautics and Astronautics, Nanjing 210016, China; 1105786873@nuaa.edu.cn (B.H.); xuchong@nuaa.edu.cn (C.X.); zengjiefan@nuaa.edu.cn (Z.F.); dr.luo@nuaa.edu.cn (D.L.); lpeng@nuaa.edu.cn (P.L.); dh_msc@nuaa.edu.cn (H.D.)

[2] Shenzhen Research Institute, Nanjing University of Aeronautics and Astronautics, Shenzhen 518000, China

* Correspondence: bingding@nuaa.edu.cn (B.D.); azhangxg@nuaa.edu.cn (X.Z.)

Characterization

The powder X-ray diffraction (XRD) (Almelo, Netherlands) patterns were recorded on Empyrean X-ray diffractor meter using Cu Kα (λ = 0.154178 nm) radiation with a scan speed of 1° min^{-1} in the 2θ range between 5–40°. Materials Studio 8.0 software was used to generate CTF structure model, and the fine XRD curve was obtained by Pawley refinement. Fourier transform infrared spectroscopy (FTIR) was obtained using Nicolet 750 Fourier (Waltham, MA, USA) Transform Infrared Spectrometer. Solid-state nuclear magnetic resonance (NMR) was carried out on a Bruker 300 MHz NMR spectrometer (Romanshorn, Switzerland) and ligand NMR data were taken in Bruker 200/400/500 MHz NMR (Romanshorn, Switzerland) spectrometer. X-ray photoelectron spectroscopy (XPS) analysis was performed on a Perkin−Elmer PHI 550 spectrometer (Waltham, MA, USA) with Al Ka (1486.6 eV) as the X-ray source. The morphology of the samples was investigated by using scanning electron microscope (SEM, Hitachi S4800) (Tokyo, Japan) coupled with an energy-dispersive X-ray spectrometer (EDX) and transmission electron microscopy (TEM, Tecnai G2 F30 S-TWIN) (Tokyo, Japan). Thermogravimetric analyses were investigated by thermo gravimetric analyzer (TGA, Pyris Diamond, PerkinElmer Thermal Analysis) (Wellesley, MA, USA) from 50 to 800 °C at a heating rate of 10 °C min^{-1} under nitrogen atmosphere. Nitrogen adsorption analyses were measured at 77 K with a Micromeritics ASAP 2460 surface characterization analyzer. The Brunauer-Emmett-Teller (BET) model was utilized to calculate the surface area. By using the non-local density functional theory (NLDFT) model, the pore size and volume were derived from the sorption curve.

Li_2S_4 adsorption experiment

The Li_2S_4 electrolyte (0.1 M) was prepared by stoichiometrically dissolving Li_2S (91.9 mg) and sulfur (194.4 mg) in 20 mL of 1,3-dioxolane/1,2-dimethoxy-ethane (DOL/DME, 1:1 in volume ratio). Five mg CTF and 5 mg CNT powder were added into 10 mL Li_2S_4 solution, respectively. Sealed bottles were stored overnight in an argon glove box.

DFT calculation

The atomic configurations and binding energies between CTF and the polysulfide anions were calculated using DFT. The method was described in our previous work[S1].

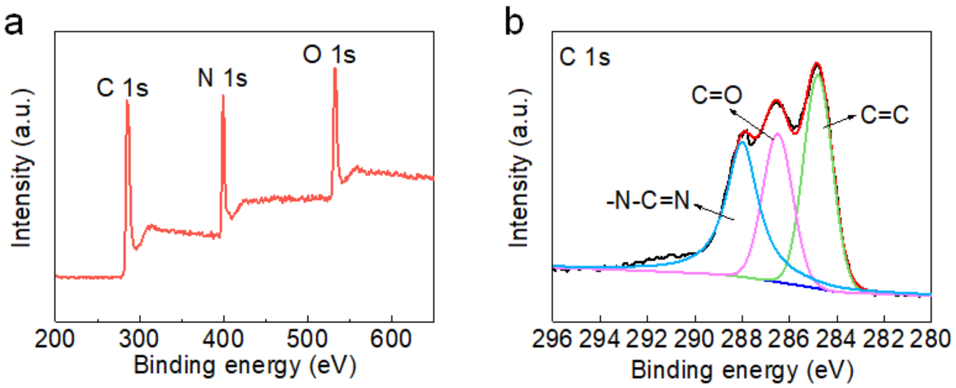

Figure S1. (**a**) XPS survey of CTF. (**b**) High-resolution C *1s* XPS spectra of CTF.

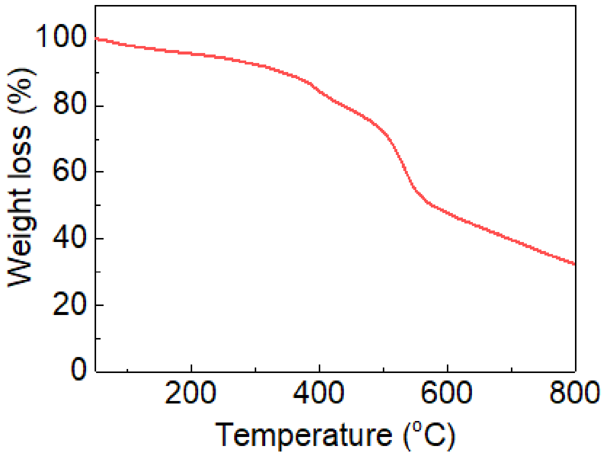

Figure S2. TGA curve of CTF measured in a nitrogen atmosphere.

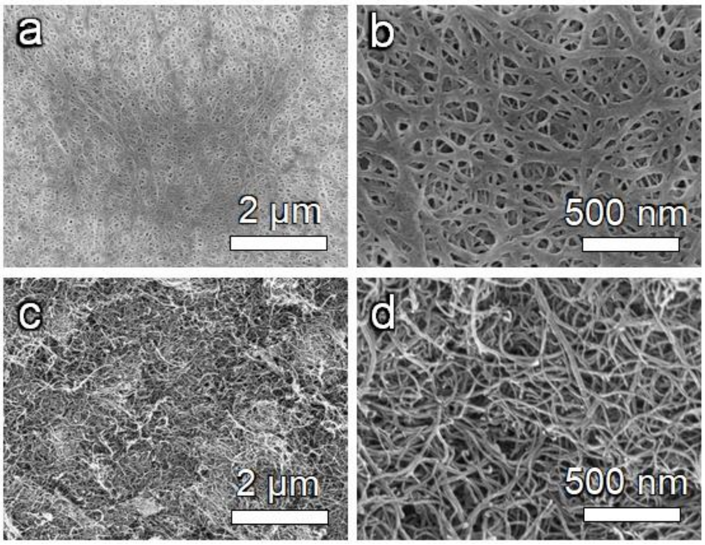

Figure S3. SEM images of (**a**, **b**) Celgard separator and (**c**, **d**) CNT/Celgard separator.

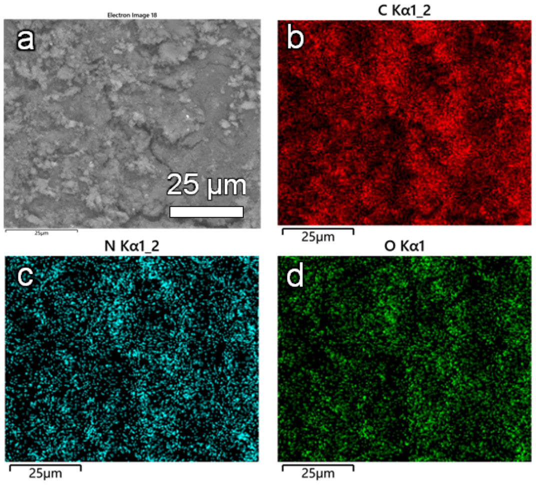

Figure S4. (**a**) Top-view SEM image and corresponding EDX mapping of (**b**) N, (**c**) O, and (**d**) S of CTF/CNT/Celgard separator.

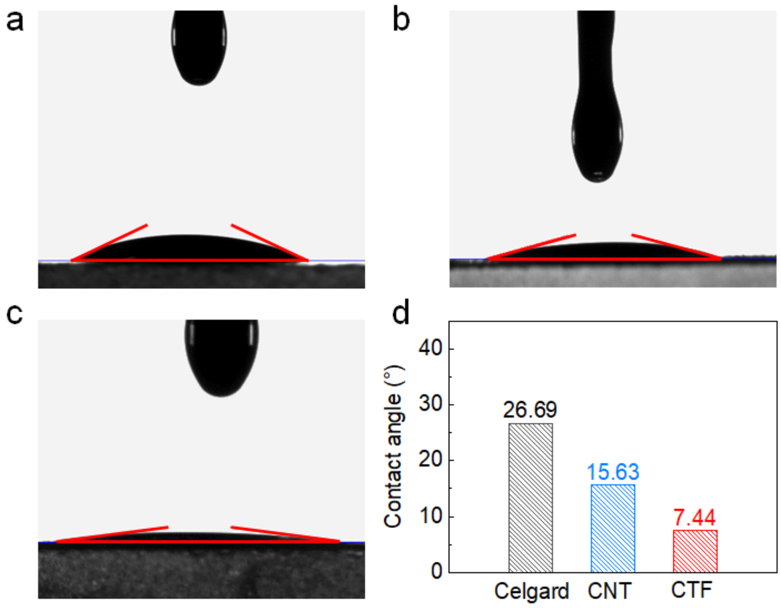

Figure S5. Contact angles of the electrolyte on (**a**) Celgard, (**b**) CNT/Celgard, and (**c**) CTF/CNT/Celgard, respectively. (**d**) Summary of the value of the contact angles.

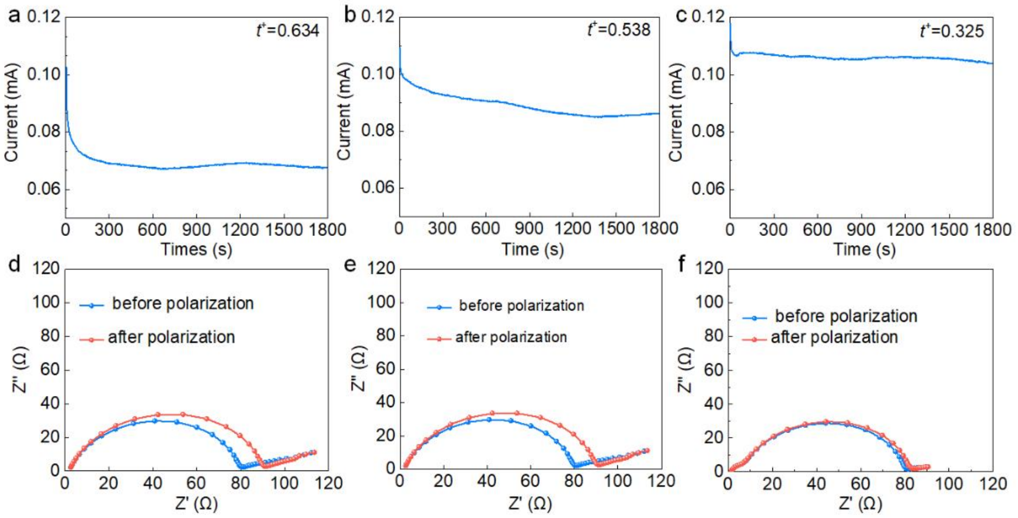

Figure S6. *I-t* curves of symmetric batteries using (**a**) CTF/CNT/Celgard separator, (**b**) CNT/Celgard separator, (**c**) Celgard separator, respectively. (**d–f**) Impedance plots estimating lithium-ion conductivity of the CTF/CNT/Celgard, CNT/Celgard and Celgard separators.

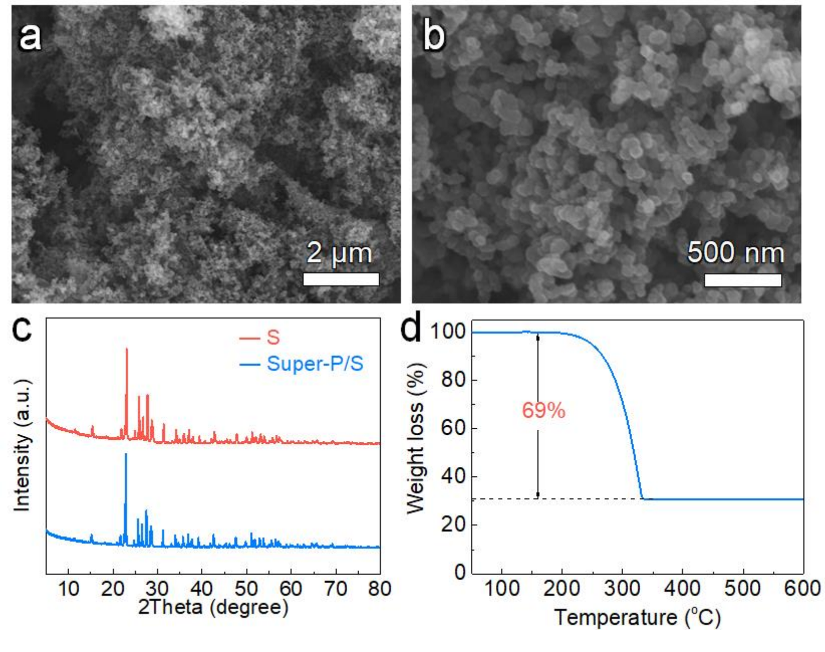

Figure S7. (**a**, **b**) SEM images of Super P/S composite. (**c**) XRD patterns of elemental sulfur and Super P/S composite. (**d**) TGA curve of Super P/S composite measured under a nitrogen atmosphere.

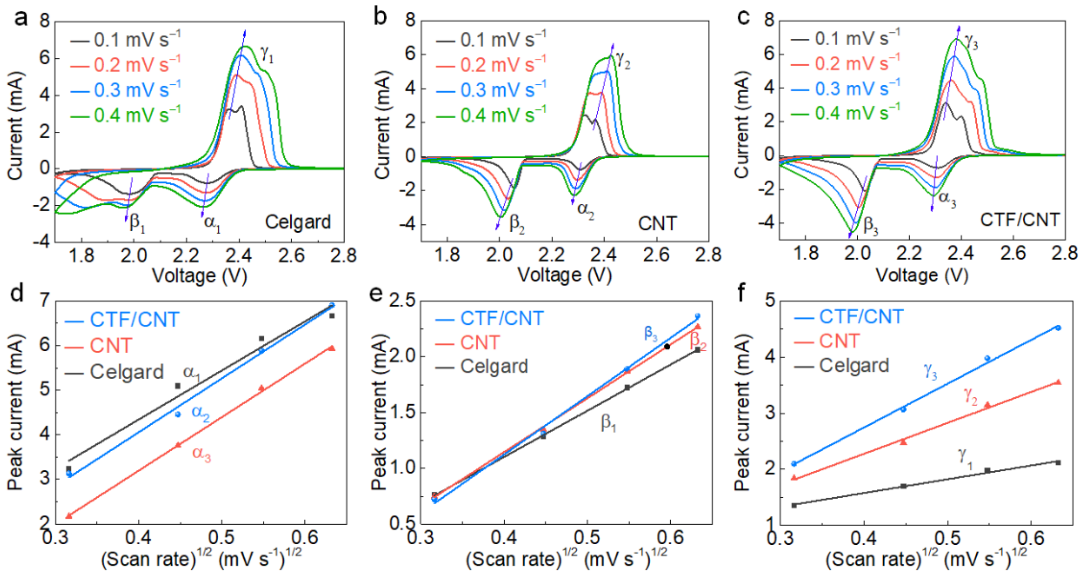

Figure S8. CV curves (scan rate: 0.1, 0.2, 0.3, 0.4 mV s^{-1}) of Li–S batteries using (**a**) Celgard separator, (**b**) CNT/Celgard separator, and (c) CTF/CNT/Celgard separator, respectively. (**d–f**) Plots of peak current *vs.* square root of scan rates of Li–S batteries using different separators, which are located at (**d**) a, (**e**) b, and (**f**) g peaks, respectively.

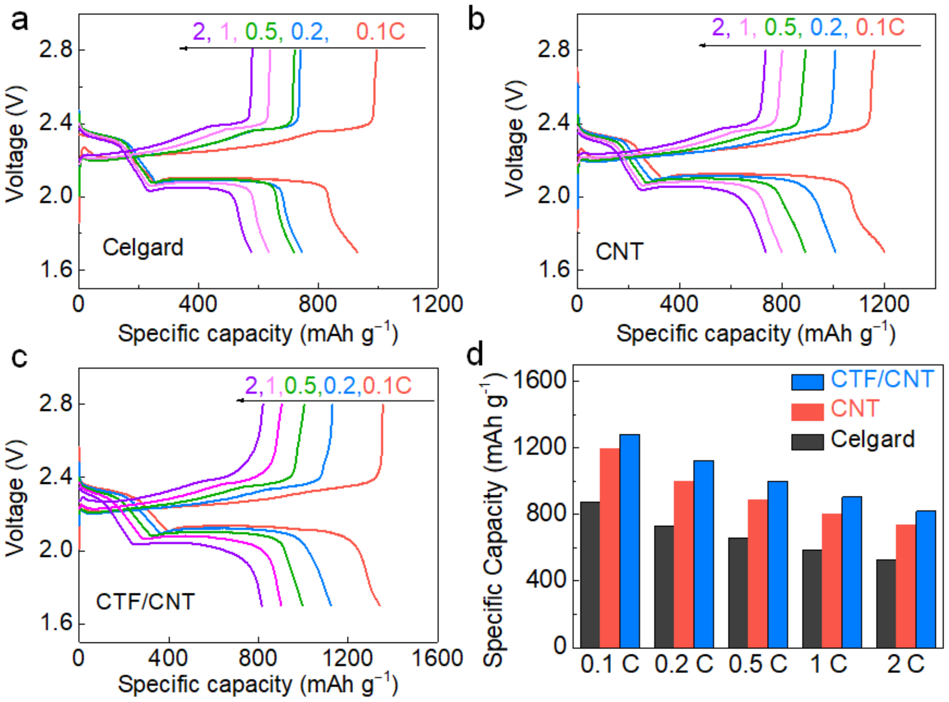

Figure S9. Galvanostatic discharge-charge profiles of the Li–S batteries with different separators at various current densities: (**a**) Celgard, (**b**) CNT/Celgard separator, and (**c**) CTF/CNT/Celgard separator. (**d**) Summary of the capacities of the Li–S batteries obtained at various current densities.

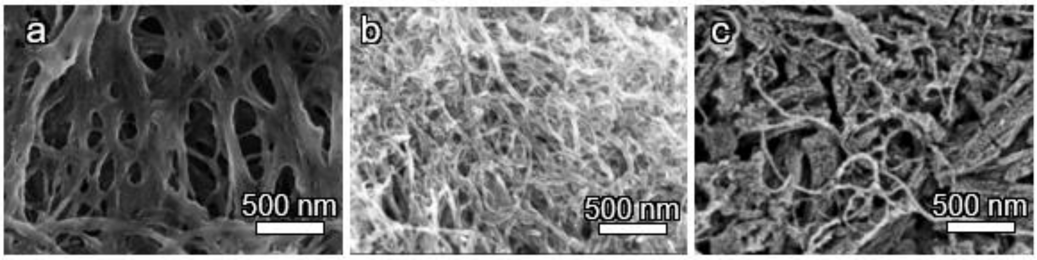

Figure S10. SEM images of the separator in the batteries after cycling: (**a**) Celgard separator, (**b**) CNT/Celgard separator, and (**c**) CTF/CNT/Celgard separator after cycling.

Table S1. Summary of lithium ion diffusion coefficient of Li–S batteries using different separators.

Separator	D(α) (cm^2 s^{-1})	D(β) (cm^2 s^{-1})	D(γ) (cm^2 s^{-1})
**Celgard**	1.4×10^{-10}	2.31×10^{-11}	8.11×10^{-12}
**CNT**	1.95×10^{-10}	3.15×10^{-11}	4.13×10^{-11}
**CTF/CNT**	1.97×10^{-10}	3.69×10^{-11}	8.27×10^{-11}

Table S2. Electrochemical performance of Li–S batteries by using various modified separators.

Functional layer on separator	Layer thickness (μm) & weight (mg cm^{-2})	Mass loading of S (mg cm^{-2})	t^+	Cycling performance	Fading rate per cycle (%)	Ref.
MoS_2	0.35 μm		0.62	401 mA h g^{-1} (0.5 C, 600 cycles)	0.083	S2
MoN-G	12 μm 0.2 mg cm^{-2}	1.0~1.2		678 mAh g^{-1} (0.5 C, 500 cycles)	0.072	S3
COF-rGO	20 μm	1.0-1.5		~600 mAh g^{-1} (1 C, 250 cycles)	0.182	S4
CTF-HUST-1	15 μm 0.14 mg cm^{-2}	2.0		700.1 mAh g^{-1} (1 C, 800 cycles)	0.052	S5
TP-BPY-COF	3 μm	1-1.5		826 mA h g^{-1} (1 C, 250 cycles)	0.173	S6
Li-CON@GN	5 μm 0.3 mg cm^{-2}	1-2	0.82	645 mAh g^{-1} (1 C, 600 cycle	0.057	S7
DMTA-COF@CNT		2.0		658 mAh g^{-1} (~1.2 C, 700 cycles)	0.070	S8
TpPa-SO_3Li	0.23 μm	5.4	0.88	639 mAh g^{-1}	0.224	S9

	0.37 mg cm^{-2}			(0.2 C, 100 cycles)		
TpPa-SO_3Li with CNT interlayer	0.23 μm	1.5		482 mAh g^{-1} (4 C, 400 cycles)	0.039	S9
CTF/CNT	5 μm 0.51 mg cm^{-2}	1.2	0.63	684 mAh g^{-1} (1 C, 400 cycles)	0.1	This work

References

[S1]. Lin, Q.; Ding, B.; Chen, S.; Li, P.; Li, Z.; Shi, Y.; Dou, H.; Zhang, X. Atomic Layer Deposition of Single Atomic Cobalt as a Catalytic Interlayer for Lithium–Sulfur Batteries. *ACS Appl. Energy Mater.* **2020**, 3, 11206–11212.

[S2]. Ghazi, Z. A.; He, X.; Khattak, A. M.; Khan, N. A.; Liang, B.; Iqbal, A.; Wang, J.; Sin, H.; Li, L.; Tang, Z. MoS_2/Celgard Separator as Efficient Polysulfide Barrier for Long-Life Lithium–Sulfur Batteries. *Adv. Mater*. **2017**, 29, 1606817.

[S3]. Tian, D.; Song, X.; Wang, M.; Wu, X.; Qiu, Y.; Guan, B.; Xu, X.; Fan, L.; Zhang, N.; Sun, K. MoN Supported on Graphene as a Bifunctional Interlayer for Advanced Li–S Batteries. *Adv. Energy Mater*. **2019**, 9, 1901940.

[S4]. Jiang, C.; Tang, M.; Zhu, S.; Zhang, J.; Wu, Y.; Chen, Y.; Xia, C.; Wang, C.; Hu, W. Constructing Universal Ionic Sieves via Alignment of Two-Dimensional Covalent Organic Frameworks (COFs). *Angew. Chem., Int. Ed.* **2018**, 57, 16072-16076.

[S5]. Shi, Q. X.; Yang, C. Y.; Pei, H. J.; Chang, C.; Guan, X.; Chen, F. Y.; Xie, X. L.; Ye, Y. S. Layer-by-layer self-assembled covalent triazine framework/electrical conductive polymer functional separator for Li–S battery. *Chem. Eng. J.* **2021**, 404, 127044.

[S6]. Xu, Q.; Zhang, K.; Qian, J.; Guo, Y.; Song, X.; Pan, H.; Wang, D.; Li, X. Boosting Lithium–Sulfur Battery Performance by Integrating a Redox-Active Covalent Organic Framework in the Separator. *ACS Appl. Energy Mater.* **2019**, 2, 5793-5798.

[S7]. Cao, Y.; Liu, C.; Wang, M.; Yang, H.; Liu, S.; Wang, H.; Yang, Z.; Pan, F.; Jiang, Z.; Sun, J. Lithiation of covalent organic framework nanosheets facilitating lithium-ion transport in lithium–sulfur batteries. *Energy Storage Mater.* **2020**, 29, 207–215.

[S8]. Wang, J.; Qin, W.; Zhu, X.; Teng, Y. Covalent organic frameworks (COF)/CNT nanocomposite for high performance and wide operating temperature lithium–sulfur batteries. *Energy* **2020**, 199, 117372.

[S9]. Cao, Y.; Wu, H.; Li, G.; Liu, C.; Cao, L.; Zhang, Y.; Bao, W.; Wang, H.; Yao, Y.; Liu, S.et al. Ion Selective Covalent Organic Framework Enabling Enhanced Electrochemical Performance of Lithium–Sulfur Batteries. *Nano Lett*. **2021**, 21, 2997–3006.
